# Red Orange: Experimental Models and Epidemiological Evidence of Its Benefits on Human Health

**DOI:** 10.1155/2013/157240

**Published:** 2013-05-02

**Authors:** Giuseppe Grosso, Fabio Galvano, Antonio Mistretta, Stefano Marventano, Francesca Nolfo, Giorgio Calabrese, Silvio Buscemi, Filippo Drago, Umberto Veronesi, Alessandro Scuderi

**Affiliations:** ^1^Section of Hygiene and Public Health, Department of G.F. Ingrassia, University of Catania, Via S. Sofia No. 85-95123, Catania, Italy; ^2^Section of Biochemistry, Department of Drug Sciences, University of Catania, Catania, Italy; ^3^Department of Biology, Piemonte Orientale University, Alessandria, Italy; ^4^Department of Internal Medicine, University of Palermo, Palermo, Italy; ^5^Section of Pharmacology and Biochemistry, Department of Clinical and Molecular Biomedicine, University of Catania, Catania, Italy; ^6^Department of Experimental Medicine, University of Rome “Sapienza”, Rome, Italy; ^7^Department of Agri-Food and Environmental Systems and Management (DIGESA), University of Catania, Catania, Italy

## Abstract

In recent years, there has been increasing public interest in plant antioxidants, thanks to the potential anticarcinogenic and cardioprotective actions mediated by their biochemical properties. The red (or blood) orange (*Citrus sinensis* (L.) Osbeck) is a pigmented sweet orange variety typical of eastern Sicily (southern Italy), California, and Spain. In this paper, we discuss the main health-related properties of the red orange that include anticancer, anti-inflammatory, and cardiovascular protection activities. Moreover, the effects on health of its main constituents (namely, flavonoids, carotenoids, ascorbic acid, hydroxycinnamic acids, and anthocyanins) are described. The red orange juice demonstrates an important antioxidant activity by modulating many antioxidant enzyme systems that efficiently counteract the oxidative damage which may play an important role in the etiology of numerous diseases, such as atherosclerosis, diabetes, and cancer. The beneficial effects of this fruit may be mediated by the synergic effects of its compounds. Thus, the supply of natural antioxidant compounds through a balanced diet rich in red oranges might provide protection against oxidative damage under differing conditions and could be more effective than, the supplementation of an individual antioxidant.

## 1. Introduction

In recent years, an increasing interest in plant antioxidants has occurred because of the potential anticarcinogenic and cardioprotective actions mediated by their biochemical properties [1–3]. The antioxidant activity of these compounds may be dependent on the number and arrangement of the hydroxyl groups and the extent of structural conjugation, as well as the presence of electron-donating and electron-withdrawing substituents in the ring structure. Due to the growing interest in these pharmacologically active components in fruits, the demand for studies conducted on specific fruit such as pigmented orange juice is increasing.

Red (or blood) orange (*Citrus sinensis* (L.) Osbeck) is a pigmented sweet orange variety typical of eastern Sicily (southern Italy), California, and Spain. The red orange is noteworthy for its excellent orange flesh color and the consistent appearance of red coloration. The red coloration of red orange is mostly caused by the presence of water-soluble anthocyanin pigments not usually found in other citrus fruits.

In this paper, we discuss the main health-related properties of red orange that include anticancer, anti-inflammatory, and cardiovascular protection activities, and the effects on health of the main constituents of the red orange (namely, flavonoids, carotenoids, ascorbic acid, hydroxycinnamic acids, and anthocyanins) and their antioxidant activity and ability to modulate some key regulatory enzymes.

## 2. History, Geographical Distribution, and Varieties of Red Oranges 

Red oranges may have originated from either China or the southern Mediterranean regions, but their exact origin is not known. It is possible that, in China, northeastern India, and southeastern Asia, *Citrus sinensis* trees were eventually transported along Asian trade routes to Africa, the Mediterranean Sea Basin, and Europe where orangeries were established. A mosaic in a Roman villa built in the first quarter of the 4th century and located about 3 km outside the town of Piazza Armerina, Sicily (southern Italy), demonstrates the presence of lime and lemon in Italy in that period of time. Citrus fruit seems to have been introduced in Sicily by Arab traders during the 7th century and cultivated as ornament until the 16th century. Spaniards introduced orange cultivation in South America in the 1500s and from there also in the United States. The first description of the red orange in Sicily was noted in the 17th century opera *Hesperides* (1646). The author described a particular kind of orange fruit (“*aurantium inducum*”), which is strongly pigmented (“*purpurei coloris medulla*”), imported to the island by a Genoese missionary from the Philippines islands.

The three most common types of red oranges are the *Tarocco*, the *Moro* (both native to Italy), and the *Sanguinello* (native to Spain). Other less common types include *Budd blood orange*, *Maltese*, *Khanpur*, *Washington Sanguine*, *Ruby Blood*, *Sanguina Doble Fina*, *Delfino*, *Red Valencia*, *Burris blood Valencia orange*, *Vaccaro blood orange*, *Sanguine grosse ronde*, *Entre Fina blood orange,* and *Sanguinello a pignu*. While also pigmented, *Cara Cara Navels* and *Vaniglia Sanguignos* have pigmentation based on lycopene, not anthocyanins like blood oranges [4]. The *Tarocco* variety is a medium-sized seedless fruit and is perhaps the sweetest and most flavorful of the three types. It is referred to as “half-blood,” because the flesh is not accentuated in red pigmentation as much as with the *Moro* and *Sanguinello* varieties. The *Moro* is the most colorful of the red oranges, referred to as “deep blood orange,” with deep red flesh ranges from orange-veined with ruby coloration, to vermilion, to vivid crimson, and nearly to black and a rind that has a bright red blush. This fruit has a distinct, sweet flavor with a hint of raspberry. The *Moro* variety is believed to have originated at the beginning of the 19th century in the citrus-growing area around Lentini (in the Province of Siracusa in Sicily) as a bud mutation of the “*Sanguinello Moscato*.” The *Sanguinello* variety, discovered in Spain in 1929, is also present in Sicily as a “full-blood” orange, close in characteristics to the *Moro*. It matures in February, but can remain on trees unharvested until April. Fruit can last until the end of May.

## 3. The Anti-Inflammatory Capacity of Red Orange Juice

 Red orange juice contains elevated quantities of various compounds including polyphenols, flavanones, anthocyanins, hydroxycinnamic acids, and ascorbic acid, and it is supposed to have a high antioxidant capacity depending on all its components (Table 1). After determining the antioxidant profile of several fresh orange juices obtained from five different *Citrus sinensis* (L.) Osbeck varieties (three pigmented varieties: *Moro*, *Sanguinello*, and *Tarocco*, and two blond varieties, *Valencia late* and *Washington navel*), the antioxidant efficiency of orange juices has been attributed, in a significant part at least, to their content of total phenols, whereas ascorbic acid seems to play a minor role [5]. *In vivo* studies conducted on healthy people has shown that red orange juice consumption determines a significant increase in plasma vitamin C, cyanidin-3-glucoside, beta-cryptoxanthin, and zeaxanthin [6]. The effect of red orange juice has been studied also in 19 subjects with increased cardiovascular risk included in a randomized, placebo-controlled, single-blind crossover study and compared with 12 healthy, nonobese control subjects in which consumption of red orange juice ameliorated endothelial functions, improving flow-mediated dilation and reducing inflammation [7].

The antioxidant activity of orange juices is related not only to structural features of phytochemicals but also to their capability to interact with biomembranes [5]. The quality control of cultivation and characteristic freshness of red oranges have demonstrated their active influence on total antioxidant activity and bioactivity of such fruit. The antioxidant capacity of red orange has been explored in two orange-based products: first, pasteurized pure juice with 40 days of shelf life, and, the second, a sterilized beverage containing minimum 12% of concentrated fruit juice [8]. Results obtained revealed that the antioxidant activity was positively related to the content of anthocyanins and the reduction of their content, typical of commercial long-shelf life juices, leading to a remarkable loss of antioxidant power. Similar results were obtained comparing both the phytochemical content (i.e., phenolics, anthocyanins, and ascorbic acid), total antioxidant activity and *in vitro* bioactivity, in terms of the protective effect obtained against oxidative damage at cellular level with organically and nonorganically grown red oranges in cell culture systems [9]. The organic orange extracts showed a higher total antioxidant activity than non-organic orange extracts due to their higher content of total phenolics, total anthocyanins, and ascorbic acid levels than the corresponding nonorganic oranges. 

Red orange intake (especially *Moro* juice) has been found to limit body weight gain, enhance insulin sensitivity, and decrease serum triglycerides and total cholesterol in mice [10, 11]. Dietary *Moro* juice markedly improved liver steatosis by inducing the expression of peroxisome proliferator-activated receptor-*α* and its target gene acylCoA-oxidase, a key enzyme of lipid oxidation. Consistently, *Moro* juice consumption suppressed the expression of liver X receptor-*α* and its target gene fatty acid synthase, and restored liver glycerol-3-phosphate acyltransferase 1 activity [10]. This action on fat accumulation has been demonstrated to be mediated by the insulin-like effect of anthocyanins cyanidin-3-O-*β*-glucoside (C3G) and its metabolite protocatechuic acid (PCA) [12]. However, the *Moro* juice antiobesity effect on fat accumulation cannot be explained only by its anthocyanin content and multiple components present in the red orange juice that might act synergistically to inhibit fat accumulation. Likewise, the anti-inflammatory effects of red orange juice do not depend only on a single component [5]. An experimental study has demonstrated that the intake of a single portion of red orange juice provides an early protection of the mononuclear red cell against oxidative DNA damage, whereas, on the contrary, no subsequent effect of a drink supplemented with the same amount of vitamin C was observed [13]. Thus, the protective effect of red orange juice was not explained by utilization of vitamin C alone. Therefore, a variety of phytochemicals contained in red oranges are assumed to be involved. 

## 4. Components of Red Orange

### 4.1. Flavonoids

Polyphenols are a group of chemical substances found in plants, especially in the genus *Citrus*, characterized by the presence of more than one phenol unit [14]. The most commonly studied polyphenols are the flavonoids, which include several thousand compounds, characterized by a common benzo-*γ*-pyrone structure [14]. Flavones, flavonols, anthocyanins and, in greater quantities, flavanones are four types of flavonoids present in *Citrus*, in concentration dependent on age of the plant and directly proportional to its mitotic activity [14]. The relatively large number of flavonoids in *Citrus* juices is a result of the many different combinations that are possible between polyhydroxylated aglycones and a limited number of mono- and disaccharides (Table 2). The most abundant flavonoid species that have been so far identified and quantified in *Citrus sinensis*, regardless of variety, is by far hesperidin, followed by narirutin and didymin [15]. These are all flavanone-O-glycosides, which account for most of the flavonoid content in juice, although a higher content has been found in red orange varieties (*Sanguinello*, *Moro*, and *Tarocco*) compared with nonpigmented variants (*Navel*, *Valencia*, and *Ovale*) [16].

The antioxidant properties of flavonoids protect by oxidative stress induced by both reactive oxygen species (ROS) and reactive nitrogen species (RNS) that have been shown to play a role in initiation and progression of CVD and atherosclerosis (Table 3). Several studies suggest that flavonoids act through several mechanisms on the NO-guanylyl cyclase pathway, endothelium derived hyperpolarizing factor(s), and endothelin-1, protecting endothelial cells inducing vasorelaxation [17]. Other evidence suggests that prevention of endothelial dysfunction, blood pressure, and oxidative stress reduction are the main actions of flavonoids [18, 19]. Their mechanism of action seems to be explained by some *in vitro* studies in which flavonoids interacted with various enzyme systems involved in cellular signaling, such as cyclo-oxygenases and lipoxygenases, phosphodiesterases, tyrosine kinases, and phospholipases [20]. These compounds also protect low-density lipoproteins (LDL) against macrophages-induced oxidation by preventing the generation of lipid hydroperoxides and to preserve R-tocopherol, an endogenous antioxidant carried in lipoproteins [21, 22]. 

Flavonoids have been demonstrated to be able to inhibit the growth of some tumors, such as colon [23], oral cancer [24], human breast cancer cells [25], lung carcinoma [26], and different melanoma cell lines, in which there have been demonstrated the antiproliferative effects but not the cytotoxic activity [27, 28]. Several flavonoids actions have been explored as possible mechanisms able to explain their anticarcinogenic effects, such as inhibition of matrix metalloproteinase (MMP) secretion, migration, invasion, and adhesion [29–33], as well as inhibition of the angiogenic process by regulating the expression of vascular endothelial growth factor (VEGF) and hypoxia-inducible factor 1*α* (HIF-1*α*) [34–36], all factors required by cancer cells to acquire metastatic properties. Some citrus flavonoids have been suggested to have potential health benefits due to their proapoptotic activity on several cancer cell lines, thus inhibiting progression of carcinogenesis [37–40].

### 4.2. Anthocyanins

Anthocyanins are a group of water-soluble plant compounds responsible for the brilliant color of fruits and flowers [41]. They are glycosylated polyhydroxy and polymethoxy derivatives of flavylium salts. The first experimental observation of anthocyanins in red orange was in 1931 by Matlack that demonstrated their presence in citrus fruits, “the red-fleshed variety, the so-called blood orange” [42] confirming a statement noticed even 15 years before [43]. Fruits subjected to thermal stress produce a greater amount of protective substances (i.e., anthocyanins) necessary to guard against unfavorable environmental conditions. The composition of Anthocyanins can be analyzed as a parameter for the assessment of authenticity and quality of foods rich in anthocyanin pigments [44, 45]. Several studies carried out on red oranges have shown that cyanidin-3-glycoside (C3G) was the main component of the fraction [46]. Some differences in anthocyanins content may occur considering different types of red oranges. Indeed, the primary anthocyanins in *Budd* blood orange, a red-colored blood orange typically grown in Florida, USA, were inverted in content, cyanidin-3-(6′′-malonylglucoside) (44.8%) followed by cyanidin-3-glucoside (33.6%). Vegetables and fruits, with special regard to pigmented oranges, such as *Moro*, *Sanguinello,* and *Tarocco* varieties, represent a natural source of anthocyanins, especially cyanidins [2]. Each cultivar shows a characteristic seasonal variation of the content of anthocyanins: the cultivar *Moro* contains the highest amount of anthocyanins, with a maximum peak at the first half of April; cultivars *Tarocco* shows the highest value in the first decade of March; cultivar *Sanguinello Nocellare* and *Sanguinello* show the lowest values of anthocyanins with the maximum values in the end of February and in the first half of March, respectively [41]. 

The antioxidant properties of anthocyanins, and especially of the C3G, depend on their radical scavenging and inhibitory effects on lipid peroxidation [2, 47] by their strong oxygen radical absorbance capacity (ORAC) and nitric oxide (NO) and cyclooxygenase inhibitory activities [48–50] (Table 3). Given the unstable nature of anthocyanins under natural conditions, it was believed that such molecules would not have antioxidant activity in living systems but many studies, instead, have demonstrated the antioxidant activity of C3G also in *in vivo* experiments [51]. The protection against oxidative damage of anthocyanins was observed as a dose-dependent decrease of ROS-mediated tissue damages after different C3G administration in living systems such as isolated Langendorff-perfused rat hearts subjected to ischemia and reperfusion [52], rat liver, kidneys, and brain [53], rabbit erythrocyte membranes, and rat liver microsomes [54]. In rats maintained on vitamin-E-deficient diets for 12 weeks in order to enhance susceptibility to oxidative damage, consumption of dietary anthocyanins significantly improved plasma antioxidant capacity, decreased the vitamin E deficiency, and enhanced hydroperoxides concentrations in liver [47]. These properties seem to be due to the cyanidin structure that allows the compound to be incorporated into the plasma membrane and cytosol of endothelial cells significantly enhancing their resistance to the damaging effects caused by several ROS-generating systems [55]. The antioxidant activity of orally administered C3G in rats was demonstrated in a model for acute oxidative stress in which C3G significantly suppressed the elevations of the liver and serum thiobarbituric-acid-reactive substance concentrations and the serum activities of marker enzymes for liver injury (GOT, GPT, and LDH) caused by hepatic ischemia-reperfusion treatment [56]. It has been suggested only in recent years that orally administered C3G is absorbed into the circulating system as a free form and subsequently metabolized to protocatechuic acid or peonidin 3-glucoside in the blood and tissues [57], and these compounds act as antioxidants in rats [58]. *In vivo* formation of protocatechuic acid following administration of C3G has been demonstrated in three species (humans, rats, and pigs), although protocatechuic acid is not retrieved in blood in 100% of cases due to differences in the experimental models (namely storage conditions, preanalytical treatments of biological samples and extraction procedure) that may affect its fugitive nature [59]. The bioavailability and biotransformation issues have to be always considered when the health efficacy of compounds from oral administration on target organs is considered. Further research should be focused not only on consequent anthocyanins effects but also on their metabolites, rather than their native forms, that reach tissues and may exert biological effects.

A number of biological activities of anthocyanins aimed at preventing cancer have been addressed [60–62]. The antimutagenic activity was demonstrated by a study on colorectal carcinogenesis inducted by 1,2-dimethylhydrazine (DMH) [63] confirming previous reports in which juice or extracts of plants containing large amounts of anthocyanins acted as inhibitors of heterocyclic amine mutagenesis [64, 65]. C3G also prevented genomic DNA damage in human fibroblast [66], hepatoma-derived cell line (Hep G2) [67], colonic adenocarcinoma (CaCo-2) [68], melanoma [69], and vulva carcinoma A431 [70] cell lines. Finally, experiments on protocatechuic acid have been demonstrated promising curative properties in its use against colon cancer [71–73].

### 4.3. Carotenoids

Fruits and vegetables are a rich source in this phytochemicals and almost 50 carotenoids can be found in the human diet [74]. The carotenoids that have been most studied in this regard are *α*-carotene, *β*-carotene, lycopene, lutein, *β*-cryptoxanthin, and zeaxanthin, almost all contained in red orange in higher quantities than in other sweet oranges (only *Cara Cara orange* demonstrated a superior contents of carotenoids compared with *Sanguinello*) [75]. Carotenoids have been implicated as important dietary nutrients having many other biological functions such as antioxidant activity, being involved in the scavenging of free radicals. Moreover, carotenoids react with singlet molecular oxygen and peroxyl radicals generated in the process of lipid peroxidation and they have been shown to protect LDL against oxidation (Table 3) [76].

In addition to their antioxidant properties, carotenoids show an array of biological effects including cardioprotective, antimutagenic and anticarcinogenic activities, involving modulation of signal transduction pathways and induction of gap-junctional communication. Dietary intakes of carotenoids have been associated with decreased risk of coronary artery disease [77], CVD [78, 79], atherosclerosis [80]. Lutein plasma changes have been associated with more promising early outcomes and decreased lipid peroxidation in subjects after ischemic stroke [81, 82], reduced risk of ischemic stroke [83]. By contrast, the risk reductions in cardiovascular events subsequent to high carotenoid intakes have appeared only to a small degree [84] or not confirmed in other studies [85, 86]. During the post-intervention follow-up, dietary supplementation with alpha-tocopherol or beta-carotene has produced neither any benefit nor harm [87]. After an average of four years of supplementation, the combination of beta carotene and vitamin A showed no benefits and may even have had an adverse effect on health, with an increased risk of death cause, lung cancer, and CVD [88]. Finally, evidence resulting from a recent randomized controlled trial on specific antioxidant supplementation was insufficient to prove the effectiveness of each of the vitamin supplements in preventing or treating cardiovascular disease [89]. To date, more information is needed to clarify the relation between the intake of single carotenoids, and the risk of heart diseases.

### 4.4. Ascorbic Acid (Vitamin C)

Vitamin C, or ascorbic acid, serves in humans as a co-factor in several important enzyme reactions and is necessary for the synthesis of collagen [90]. Due to the incapacity to synthesize vitamin C, humans require it from natural sources through supplements to the ordinary diet. Lack of vitamin C results in scurvy, a pathological condition characterized by friable vessels, especially in capillary tissues that are most likely to rupture, and also petechial hemorrhages and ecchymosis due to a deficit of collagen synthesis and secretion to form the extracellular matrix or part of the basement membrane [90]. The vitamin C content of red oranges is in the range of 32 to 42 mg per 100 mL, with the highest levels found in the *Sanguinello* varieties, followed by *Cara Cara* navels and *Moro *(the US recommended daily allowance for vitamin C is set at 75 mg for women and 90 mg for men) [46].

At physiological concentrations, vitamin C is a potent-free radical scavenger in plasma, protecting cells against oxidative damage caused by ROS (Table 3) [91]. The antioxidant property of ascorbic acid is attributed to its ability to reduce potentially damaging ROS, forming, instead, resonance-stabilized and relatively stable ascorbate free radicals [92]. These antioxidant capacities lead to numerous effects of vitamin C on vascular bed, such as induction of endothelial-dependent artery dilation and increase of blood flow [93], attenuation of *in vitro* and *in vivo* LDL-oxidation [94–96], and protection of human vascular smooth muscle cells against apoptosis [97, 98]. In light of the several benefits of vitamin C on endothelial cell proliferation, function, and viability, it is plausible that increases in plasma and cell content of such vitamins might help to prevent, delay, or stabilize early endothelial dysfunction associated with atherosclerosis.

Several studies hypothesized that the anti-inflammatory properties of Vitamin C may reduce the incidence of many malignancies in humans due to a number of cytoprotective functions under physiological conditions, including prevention of DNA mutation induced by oxidation by neutralizing potentially mutagenic ROS [99–102]. Indeed, consumption of vitamin-C-rich foods has been found to be inversely related to the level of oxidative DNA damage *in vivo *[103]. However, the inconsistency of the vitamin C cancer correlation and lack of validated mechanistic basis for its therapeutic action underline its potential role as a preventive rather than therapeutic drug.

### 4.5. Hydroxycinnamic Acids

Hydroxycinnamic acids (hydroxycinnamates) are a class of polyphenols having a C6-C3 skeleton. These compounds are hydroxy derivatives of cinnamic acid. In the category of phytochemicals that can be found in red orange, the most common are caffeic, p-coumaric, ferulic, and sinapic [104]. Free and bound ferulic acid represent the major component in all cases, followed by p-coumaric acid, sinapic acid, and caffeic acid. However, hydroxycinnamic acids have been found to be more abundant in red orange than in blond juices. Ferulic acids and caffeic acid are among the most studied hydroxycinnamic acids (Table 3).

Ferulic acid is an abundant phenolic phytochemical found in plant cell wall components such as arabinoxylans as is found, for instance, in covalent side chains. It is related to *trans*-cinnamic acid. As a component of lignin, ferulic acid is a precursor in the manufacture of other aromatic compounds. It has been demonstrated to be successfully employed as topical protective agents against UV radiation-induced skin damage after *in vitro* and *in vivo* evaluation [105], often in combination with other antioxidants such caffeic acid [106] and vitamin C [107]. Its mechanisms of action may depend on preventing DNA damage and restoring antioxidant status and histopathological changes [108]. The anticarcinogenic properties of ferulic acid have been studied on mammary [109], buccal pouch [110], colon [111], and skin [112] carcinogenesis in experimental models findings showing that oral administration of ferulic acid significantly prevented the onset of tumors and the biochemical and molecular abnormalities. Although the exact chemopreventive mechanism of the ferulic acid is unclear, its antigenotoxic and antioxidant potentials as well as modulatory effect on phase II detoxification cascade could play a possible role [109].

Caffeic acid is an organic compound that is classified as a hydroxycinnamic acid. This acid consists of both phenolic and acrylic functional groups. It is found in all plants because it is a key intermediate in the biosynthesis of lignin, one of the principal sources of biomass [113]. The caffeic acid phenethyl ester has been found to be a potent free radical scavenger [114] and studied for its antioxidant capacities in several experimental rat models on renal impairment [115–117], retinal oxidative stress [118], myocardial oxidative stress [119], age-related vascular remodeling and cardiac damage [120], and at the same time helping to prevent the metabolic consequences in diabetes mellitus [121, 122], erythrocyte membrane ischemia/reperfusion injury [123], cerebral damage induced by ischemia reperfusion [124], and oxidative stress [125]. The mechanisms by which caffeic acid phenethyl ester exerts its anti-inflammatory action seems to depend on its effect on lipid peroxidation (LPO) and the inhibition of antioxidant enzymes such as superoxide dismutase and catalase [126]. Moreover, caffeic acid phenethyl ester has also been shown to cause dose-dependent suppression of prostaglandins synthesis suppressing the expression of cyclooxygenase-2 in cultured human oral epithelial cells and in an animal model of acute inflammation [127] and in the protection of mice from lethal endotoxin shock. It also inhibits lipopolysaccharide-induced cyclooxygenase-2 and inducible NO synthase expression in RAW 264.7 macrophages via the p38/ERK and NF-*κ*B pathways, therefore providing mechanistic insights into the anti-inflammatory and chemopreventive actions of caffeic acid phenethyl ester in macrophages [128].

## 5. Conclusions

On the basis of these findings, evidence has shown that red oranges demonstrate both potent antioxidant activity and also cytoprotective effects that reflect their substantial role in preventing chronic pathological conditions such as cardiovascular diseases and in many forms of cancers. A synergic action between organic farming and social activities may amplify the advantages and reciprocal benefits in order to obtain a “social and environmental sustainability” and spread consumption of healthy products [129, 130]. The supply of natural antioxidant compounds through a balanced diet rich in red oranges might provide protection against oxidative damage under different conditions and could be more effective than supplementation of an individual antioxidant. 

## Figures and Tables

**Table 1:** Main components of red orange fruit.

Food components	Value
Proximates	
Energy, recalculated, kJ	144
Energy, recalculated, kcal	34
Total protein, g	0.7
Animal protein, g	0.0
Vegetable protein, g	0.7
Total fat, g	0.2
Animal fat, g	0.0
Vegetable fat, g	0.2
Cholesterol, mg	0
Available carbohydrates, g	7.8
Starch, g	0.0
Soluble carbohydrates, g	7.8
Dietary total fibre, g	1.6
Alcohol, g	0.0
Water, g	87.2
Minerals and traces elements	
Iron, mg	0.2
Calcium, mg	49
Sodium, mg	3
Potassium, mg	200
Phosphorus, mg	22
Zinc, mg	0.20
Water soluble vitamins	
Vitamin B1, thiamin, mg	0.06
Vitamin B2, riboflavin, mg	0.05
Vitamin C, mg	50
Niacin, mg	0.20
Vitamin B6, mg	0.10
Total folate, *µ*g	31
Retinol eq., *µ*g	71
Retinol, *µ*g	0
*β*-Carotene eq., *µ*g	426
Vitamin E, *α*-tocopherol eq., mg	0.24
Vitamin D, *µ*g	0.00
Fatty Acids	
Saturated fatty acids, g	0.03
Monounsaturated fatty acids, g	0.04
Oleic acid, g	0.03
Polyunsaturated fatty acids, g	0.04
Linoleic acid, g	0.03
Linolenic acid, g	0.01

Source: European Institute of Oncology (EIO) database at http://www.ieo.it/bda2008/homepage.aspx.

**Table 2:** Main polyphenols contained in red orange juice.

Polyphenol group	Single component	Mean content	min	max	SD
Flavonoids					
Anthocyanins	Cyanidin 3-O-glucoside	1.41 mg/100 mL	0.28	4.03	1.32
	Cyanidin 3-O-(6′′-malonyl-glucoside)	1.76 mg/100 mL	0.37	3.86	1.30
Flavanones	Hesperidin	43.61 mg/100 mL	18.00	66.50	17.98
	Narirutin	4.80 mg/100 mL	2.90	6.46	1.28
	Didymin	2.43 mg/100 mL	0.89	3.53	1.27
Flavones	Sinensetin	0.26 mg/100 mL	0.26	0.26	0.00
	Nobiletin	0.31 mg/100 mL	0.31	0.31	0.00
	Tangeretin	0.04 mg/100 mL	0.04	0.04	0.00
Flavonols	3-Methoxynobiletin	0.08 mg/100 mL	0.08	0.08	0.00

Phenolic acids					
Hydroxycinnamic acids	Cinnamoyl glucose	1.50 mg/100 mL	0.41	3.74	1.50
	Cinnamic acid	0.02 mg/100 mL	7.00*e* − 03	0.06	0.03
	p-Coumaric acid	2.92 mg/100 mL	1.24	4.46	0.84
	Caffeic acid	0.88 mg/100 mL	0.44	1.51	0.34
	Ferulic acid	4.40 mg/100 mL	3.16	6.37	0.75
	Sinapic acid	1.74 mg/100 mL	1.01	3.59	0.63

Source: phenol-explorer at http://www.phenol-explorer.eu/.

**Table 3:** Name, effect, and mechanisms of action of main components of red orange.

Food components	Effect	Mechanisms of action
Flavonoid	Anti-inflammatory	Modulate apoB secretion and cellular cholesterol; help cholesterol levels by raising HDL and lowering LDL cholesterol
	Antioxidant	Stimulate endothelial NO synthase; normalize lipid peroxidation markers
	Antiaggregation	Inhibit TxA2-mediated responses and dense granule secretion
	Anticarcinogenic	Promote apoptosis in human pre-B NALM-6 cells and colon cancer cells; inhibit HIF-1*α* and VEGF expression in ovarian cancer and in lung cancer
	Antiproliferative	Inhibit the COX-2 and MMPs in lung, prostate, and hepatocellular carcinoma cells; inhibited the proliferation of MCF-7 human breast cancer cells and testosterone-induced proliferation of LNCaP cells; inhibit lung colonization by melanoma and sarcoma cell line; inhibit formation of new blood vessels in human breast cancer cells

Anthocyanins	Antioxidant	Protect biomembranes from peroxidation by trapping peroxyl radicals in the cytosol; chelate metal ions like Cu2; ability in chelating metal ions like Cu2; form an ascorbic acid metal-anthocyanin complex (copigment)
	Antimutagenicity	Form a cyanidin-DNA copigmentation complex; inhibit the reverse mutation induced by heterocyclic amines in microsomal activation systems
	Growth inhibition	Inhibit the tyrosine kinase activity of the EGFR and the activation of the GAL4-Elk-1 fusion protein

Carotenoids	Antioxidant	React with singlet molecular oxygen and peroxyl radicals

Vitamin C	Blow-flow increase	Enhance generation of NO; reduce nitrite; stabilize atherosclerotic plaques (due to effect on collagen synthesis)
	Antioxidant	Reduce the affinity of LDL-bound apoB protein for transition metal ions; quench aqueous ROS and RNS, decreasing their bioavailability in the plasma; reduce potentially damaging ROS, forming resonance-stabilized and relatively stable ascorbate free radicals; attenuate LDL-oxidation and protection of human vascular smooth muscle cells against apoptosis

Hydroxycinnamic acids	Antioxidant	Effect on phase II detoxification cascade; inhibit of superoxide dismutase and catalase; suppress of PG synthesis and cyclooxygenase-2
	Anticarcinogenic	Prevent the tumor onset and protect the biochemical and molecular abnormalities in mammary, buccal pouch, colon, and skin cancers

EGFR: epidermal growth-factor receptor; HDL: high-density lipoproteins; HIF-1*α*: hypoxia-inducible factor 1*α*; LDL: low-density lipoproteins; MMP: matrix metalloproteinase; NO: nitric oxide; PG: prostaglandins; RNS: reactive nitrogen species; ROS: reactive oxygen species.
